# Guillain–Barré Syndrome Associated with COVID-19 in a Japanese Male

**DOI:** 10.1155/2022/6837851

**Published:** 2022-10-22

**Authors:** Yoshiki Esa, Yuta Kajiyama, Misako Kaido, Yushi Watanabe, Harutoshi Fujimura, Iwao Gohma, Kenichi Takahashi, Junya Kobayashi

**Affiliations:** ^1^Department of Neurology, Sakai City Medical Center, Sakai, Japan; ^2^Department of Clinical Genetics, Sakai City Medical Center, Sakai, Japan; ^3^Department of Respiratory Medicine, Sakai City Medical Center, Sakai, Japan; ^4^Department of Respiratory Medicine, Kishiwada City Hospital, Kishiwada, Japan

## Abstract

April 2021 saw a widespread outbreak of severe acute respiratory syndrome coronavirus 2 (SARS-CoV-2) in Osaka, Japan. We encountered the case of a 52-year-old man who had Guillain–Barré syndrome associated with coronavirus disease 2019 (COVID-19). After the relief of the respiratory symptoms owing to COVID-19, the patient experienced muscle weakness, which spread from his fingers to his extremities, and was unable to walk. Further examinations revealed mild protein elevation in the cerebrospinal fluid. In addition, nerve conduction studies showed demyelinating polyneuropathy, leading to the diagnosis of Guillain–Barré syndrome. After the administration of intravenous immunoglobulin and intravenous methylprednisolone, his symptoms drastically improved, and he was able to walk unaided 21 days after the onset of symptoms. On day 40, the patient was discharged with minimal muscle fatigue. Because Guillain–Barré syndrome associated with COVID-19 is expected to have a good prognosis, early diagnosis and treatment are important. Therefore, Guillain–Barré syndrome should be considered as a possible factor for muscle weakness during and after COVID-19 treatment.

## 1. Introduction

April 2021 saw a major outbreak of severe acute respiratory syndrome coronavirus 2 (SARS-CoV-2) in Osaka, Japan. From March to June 2021, there were more than 50,000 patients with coronavirus disease 2019 (COVID-19) in Osaka prefecture, which was the highest number of cases reported in Japan during that period. COVID-19 is widely recognized to present not only with respiratory symptoms but also with various neuromuscular complications. Several polyneuropathies such as Guillain–Barré syndrome and Miller-Fisher syndrome have been reported as neurological complications of COVID-19 [1]. In 2020, numerous case reports of Guillain–Barré syndrome during or after COVID-19 treatment were reported worldwide, and the rate of occurrence was assumed to be 15 per 100,000 patients [2]. It was also known that Guillain–Barré syndrome had regional variations in clinical features and outcomes [3]. Nevertheless, only a few cases of Guillain–Barré syndrome associated with COVID-19 were reported in Japan. Here, we report a case of Guillain–Barré syndrome after COVID-19, in which the clinical symptoms progressed very rapidly but drastically improved after immunological treatment.

## 2. Case Presentation

A 52-year-old man with a medical history of allergic rhinitis, reflux esophagitis, and asthma maintained on a combination of inhaled salmeterol xinafoate and fluticasone propionate, was admitted to our hospital with symptoms of Guillain–Barré syndrome after being treated for COVID-19. He worked as frontline healthcare professional for the COVID-19 response in his hospital. He received his first tozinameran (Pfizer-BioNTech COVID-19 Vaccine; Comirnaty) vaccination at the end of March 2021. In early April, he developed a fever, olfactory disturbance, and dyspnea. Two days after the onset of these symptoms, he was diagnosed with COVID-19 via SARS-CoV-2 ribonucleic acid (RNA) detection in a nasopharyngeal swab. He then began experiencing respiratory impairment with decreased oxygenation and was admitted to a hospital, where he was treated with Remdesivir and dexamethasone, which resolved his fever and dyspnea. However, 10 days after the onset of COVID-19 (when the first symptoms presented), he experienced weakness in his fingers. His muscle weakness progressed rapidly to both of his lower limbs during the following 2 days, and he had difficulty in standing and walking. He was then transferred to the COVID-19 isolation ward in our hospital. On admission (day 2), he had no fever or respiratory difficulties. However, he showed symmetrical muscle weakness in the neck and extremities; specifically of grade 3 in his upper limbs and grade 4 in his lower limbs, according to the Medical Research Council (MRC) scale. The tendon reflexes of the extremities were also decreased. No abnormality was found in the cranial nerves testing, including the gag reflex. No sensory or autonomic disturbances were present, except for hyperhidrosis. Hematological tests showed a positive inflammatory reaction (CRP 1.56 mg/ml), but no other abnormalities were detected in the blood counts or biochemistry. The antiganglioside antibody test measured by ELISA showed a mildly positive IgG anti-GM3 antibody. Cerebrospinal fluid (CSF) examination showed a normal protein level (25 mg/dL) and cell count (1/mm^3^) at admission, however, on day 14, a mildly elevated protein level (55.1 mg/dL) and a cell count of up to 9/mm^3^ were observed. SARS-CoV-2 RNA with the N501Y mutation was detected in the nasopharyngeal swab but was negative in the CSF. Nerve conduction studies (NCS) on admission showed prolonged distal latency (DL) in the left median nerve and posterior tibial nerve. Though compound muscle action potential (CMAP) elicited at distal stimulations was within the normal range, proximal CMAP was eliminated in the posterior tibial nerve and decreased in the median nerve. The left median and sural nerves showed decreased sensory conduction velocity (SCV) and normal sensory nerve evoked potentials (SNAPs) (Table 1).

**Figure 1:**
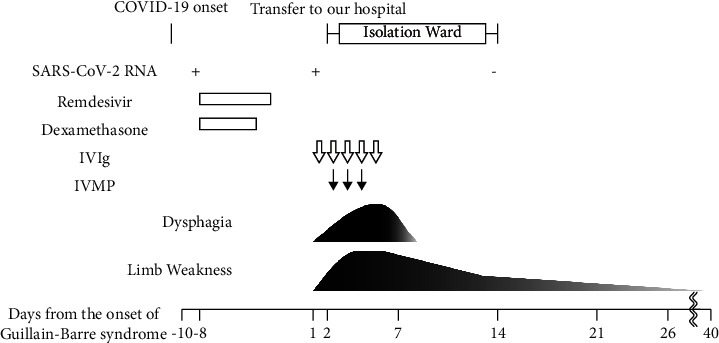
Clinical course of the disease. The diagram shows the clinical severity of dysphagia and weakness of the limbs from the onset of Guillain–Barré syndrome on day 1. The arrows indicate Guillain–Barré syndrome treatment with intravenous immunoglobulin (white) and intravenous methylprednisolone (black). On day 7, he showed severe muscle weakness in his neck and extremities, dysphagia, and respiratory difficulties. Thereafter, each symptom improved, and he was discharged on day 40 with minimal muscle fatigue. Abbreviations: IVIg, intravenous immunoglobulin; IVMP, intravenous methylprednisolone; SARS-CoV-2 RNA, severe acute respiratory syndrome coronavirus 2 ribonucleic acid.

Based on these findings, he was diagnosed with Guillain–Barré syndrome according to the Brighton criteria with level 2 diagnostic certainty. We also considered critical illness polyneuropathy due to COVID-19 as a differential diagnosis. However, we determined that Guillain–Barré syndrome was more appropriate because motor symptoms appeared after COVID-19 symptoms with an improvement of serum inflammatory markers over time. He was administered intravenous immunoglobulin (400 mg/kg/day for 5 days) on the day of admission. His muscle weakness progressed rapidly, and he developed mild difficulty in swallowing on the third day. Additional intravenous methylprednisolone (1000 mg/day for 3 days) was administered, considering the complications of a prolonged proinflammatory state with COVID-19. On day 7, when his symptoms were at their worst, he was almost completely paralyzed with MRC grade 1 (trace muscle activation, such as a twitch, without achieving full range of motion) muscle weakness in his neck and extremities, dysphagia, and respiratory difficulties. His symptom severity was at grade 4 on the Hughes functional grading scale, and the modified Erasmus Guillain–Barré syndrome outcome score was 10. On day 14, he was released from isolation and underwent further rehabilitation. He was able to walk on parallel bars on day 15, walk unaided on day 21, and was transferred to a rehabilitation hospital on day 26. He was discharged on day 40 with minimal muscle fatigue (Figure 1). The NCS on day 14 revealed an improvement in DL and CMAP. However, lower MCV in the left medial nerve and posterior tibial nerve persisted. Prolonged *F*-wave latency in the left ulnar nerve and posterior tibial nerves, as well as the conduction block in the left posterior tibial nerve, were observed. On day 48, CMAP was further improved, and the conduction block disappeared. However, the decreasing SNAP of the left median and sural nerves persisted until day 48 (Table 1).

## 3. Discussion

We report a case of Guillain–Barré syndrome that developed after COVID-19. The symptoms progressed rapidly, with prolonged distal latency on NCS immediately after symptom onset. However, both clinical and electrophysiological findings improved shortly after immunological treatments.

It is reported that Guillain–Barré syndrome associated with COVID-19 develops on average 12.1 days after COVID-19 and is characterized by demyelination, albuminocytological dissociation, absence of SARS-CoV-2 RNA in CSF, and a good response to immunological treatments [2, 4]. In our case report, Guillain–Barré syndrome developed 10 days after COVID-19 onset. Although the rapid progression and severity of the symptoms implied a poor prognosis, the progression of the disease stopped on day 7 after Guillain–Barré syndrome onset due to immunological treatment. So far, three cases of Guillain–Barré syndrome associated with COVID-19 have been reported in Japan; one case of axonal neuropathy, [5] one of acute inflammatory demyelinating polyneuropathy, [6] and an unidentified case [7]. In these cases, Guillain–Barré syndrome developed 16 to 20 days after the onset of COVID-19. Although clinical manifestations varied widely among patients with Guillain–Barré syndrome associated with COVID-19, all patients showed good improvement with or without immunotherapy (Table 2).

**Figure 2:**
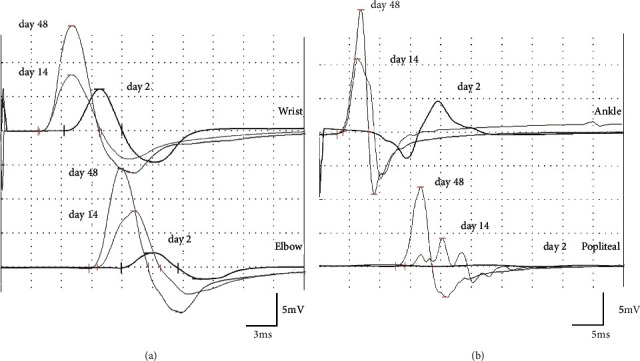
Serial nerve conduction tracings in the case. Serial recordings of compound muscle action potentials (CMAP) of the left median nerve (a) and the left posterior tibial nerve (b) on days 2, 14, and 48. Prolonged distal latencies (DL) were shown in the left median nerve and posterior tibial nerve on day 2. Distal CMAP was within the normal range; however, proximal CMAP was eliminated in the posterior tibial nerve and decreased in the median nerve. DL and CMAP improved over time on day 14 and day 48.

The present case showed prolonged DL and CMAP reduction between distal and proximal stimuli in the left median nerve and posterior tibial nerve. These findings improved on day 14 and recover on day 48 without remyelination processes, indicating an acute motor axonal neuropathy (AMAN) with reversible conduction failure [8] (Figure 2) Many cohort studies were published on Guillain–Barré syndrome after COVID-19, however, these studies included very few cases from the East Asian population. Considering current and previous reports, there seem to be cases of AMAN as well as demyelinating neuropathy in Japan. As with classic Guillain–Barré syndrome, AMAN may be more common in the Japanese population. Therefore, further epidemiological investigations are needed in Japanese and Asian populations.

The pathogenesis of Guillain–Barré syndrome associated with COVID-19 is thought to involve immune-mediated mechanisms rather than direct damage due to SARS-CoV-2 [9]. In this case, anti-GM3 antibodies were detected. Antibodies against the common epitopes of GM3, GD1a, and GT1b have been reported to be associated with the pathogenesis of bulbar palsy and respiratory failure [10]. There have been reports of Guillain–Barré syndrome with anti-GM3 antibodies, but these cases were copositive with other antiganglioside antibodies, such as GM1, GD3, and GT3 [11, 12]. Thus, although the pathological significance of pure anti-GM3 antibodies is unclear, it is possible that anti-GM3 or coexisting antiganglioside antibodies were involved in the development of Guillain–Barré syndrome in this case.

In addition, pathological findings in patients with COVID-19 revealed the expression of myxovirus resistance protein A (MxA), which is expressed in the capillaries around the peripheral nerves affected by type I interferon as an antiviral cytokine. This suggests that a COVID-19-induced cytokine storm is involved in peripheral nerve injury [13]. Corticosteroids are sometimes administered to treat central nervous system inflammatory complications associated with COVID-19 [14]. There is a possibility that corticosteroid therapy is effective for peripheral nerve inflammation. In this case, we administered corticosteroid therapy combined with IVIG. Peripheral polyneuropathy following systemic inflammatory reactions is also known as critical illness polyneuropathy. For its treatment, it is suggested that the aggressive management of medical conditions and glycemic control is vital [15]. In such a scenario, the use of glucocorticoids may be refrained. Further studies are needed to assess the effectiveness of this combined therapy.

Although pathological associations between COVID-19 and Guillain–Barré syndrome development have been suggested, epidemiological data did not show an increased incidence of Guillain–Barré syndrome per patient with COVID-19 in 2020 [16]. Therefore, there may be a weak causal relationship between SARS-CoV-2 and Guillain–Barré syndrome, as opposed to the pattern observed with the Zika virus [17].

The incidence of COVID-19 remains high in Japan in 2022. Although Guillain–Barré syndrome associated with COVID-19 is expected to have a good prognosis, early diagnosis and treatment are still important. In conclusion, Guillain–Barré syndrome should be considered as a differential diagnosis when muscle weakness is suspected during or after COVID-19 treatment.

## Figures and Tables

**Table 1:** Results of the nerve conduction studies during the clinical course.

	Distal latency (ms)	CMAP distal (mV)	CMAP proximal (mV)	MCV (m/s)	*F*-wave minimal latency (ms)	SNAP (uV)	SCV (m/s)
*Median L*							
Day 2	6.26	6.16	2.25	38.5	NA	21.90	25.6
Day 14	3.8	8.8	8.8	38.0	34.9	13.8	48.2
Day 48	4.1	15.4	14.8	51.4	30.9	15.5	60.0

*Ulnar L*							
Day 2	4.04	6.15	3.14	42.8	NA	NA	NA
Day 14	2.8	9.2	5.9	55.8	37.3	17.3	53.6
Day 48	2.9	11.8	11.2	54.1	32.0	21.5	55.6

*Tibial L*							
Day 14	4.4	11.0	4.5	34.1	61.4		
Day 48	3.3	18.6	12.2	45.4	53.7		

*Sural L*							
Day 2						10.70	31.2
Day 14						7.9	45.8
Day 48						8.4	50.0

Abbreviations: L, left; CMAP, compound motor action potential; MCV, motor conduction velocity; SNAP, sensory nerve action potential; SCV, sensory conduction velocity; NA, not available.

**Table 2:** Demographics of previous and present cases of Guillain-Barré syndrome associated with COVID-19 in Japan.

Case	Age	Sex	Clinical feature	GBS onset from the onset of COVID-19	GBS onset from COVID-19 recovery	GBS electrophysiological subtype	Anti-ganglioside antibody	CSF protein (mg/dL)	Immunological treatment
Wada et al. [7]	69	Male	Absence of cough reflex, muscle weakness, areflexia	Not described	Not described	Not described	Anti-Gal-C antibody	202	IVIg
Hirayama et al. [5]	54	Female	Muscle weakness, numbness, and areflexia in the lower limb	20	Approximately 1 week	Axonal type	Negative	Normal level	No treatment
Kakumoto et al. [6]	22	Male	Muscle weakness, hypesthesia, dysarthria, dysphagia, left-sided facial numbness, and dysautonomia	16	6	AIDP	Negative	307	IVIg
Present case	52	Male	Muscle weakness, dysphagia, areflexia, hyperhidrosis	10	1	AIDP	Anti-GM3 antibody	55	IVIg, IVMP

Abbreviations: GBS, Guillain–Barré syndrome; CSF, cerebrospinal fluid; AIDP, acute inflammatory demyelinating polyneuropathy; IVIg, intravenous immunoglobulin; IVMP, intravenous methylprednisolone.

## Data Availability

The data used to support the findings of this study are available from the corresponding author upon request.
